# The neuroendocrine pathways and mechanisms for the control of the reproduction in female pigs

**DOI:** 10.1590/1984-3143-AR2021-0063

**Published:** 2021-12-10

**Authors:** Shuang Zhao, Zongyi Guo, Wei Xiang, Pingqing Wang

**Affiliations:** 1 College of Bioengineering, Chongqing University, Chongqing, P. R. China; 2 Chongqing Academy of Animal Sciences, Chongqing, P. R. China; 3 School of Advanced Agriculture and Bioengineering, Yangtze Normal University, Fuling of Chongqing, P. R. China

**Keywords:** pig, reproduction, neuroendocrine, GnRH neuron, RFamide-related peptide-3

## Abstract

Within the hypothalamic-pituitary-gonad (HPG) axis, the major hierarchical component is gonadotropin-releasing hormone (GnRH) neurons, which directly or indirectly receive regulatory inputs from a wide array of regulatory signals and pathways, involving numerous circulating hormones, neuropeptides, and neurotransmitters, and which operate as a final output for the brain control of reproduction. In recent years, there has been an increasing interest in neuropeptides that have the potential to stimulate or inhibit GnRH in the hypothalamus of pigs. Among them, Kisspeptin is a key component in the precise regulation of GnRH neuron secretion activity. Besides, other neuropeptides, including neurokinin B (NKB), neuromedin B (NMB), neuromedin S (NMS), α-melanocyte-stimulating hormone (α-MSH), Phoenixin (PNX), show potential for having a stimulating effect on GnRH neurons. On the contrary, RFamide-related peptide-3 (RFRP-3), endogenous opioid peptides (EOP), neuropeptide Y (NPY), and Galanin (GAL) may play an inhibitory role in the regulation of porcine reproductive nerves and may directly or indirectly regulate GnRH neurons. By combining data from suitable model species and pigs, we aim to provide a comprehensive summary of our current understanding of the neuropeptides acting on GnRH neurons, with a particular focus on their central regulatory pathways and underlying molecular basis.

## Introduction

The reproductive function of pigs is controlled by complex regulatory networks, which integrate peripheral and internal cues and impinge at the brain centers driving the reproductive axis. GnRH is synthesized in a small subset of hypothalamic neurons, which form the final common pathway for the central control of reproduction (Herbison 2016). They integrate steroidal, lactational, hunger, stress, satiety, circadian, odorant, and pheromone signals (Spergel 2019). These signals are conveyed to a large extent by neuropeptides directly and/or indirectly, as well as by conventional neurotransmitters, gaseous transmitters, gliotransmitters, and other factors. GnRH neurons synthesize and secrete GnRH in a pulsatile manner from axon terminals in the median eminence (ME) into the hypothalamo-hypophyseal circulation through which it is transported to the anterior pituitary gland. By binding to specific receptors (gonadotropin-releasing hormone receptors, GnRHRs) on pituitary gonadotropin cells, GnRH stimulates the biosynthesis and the release of two gonadotropins (luteinizing hormone, LH; follicle-stimulating hormone, FSH). LH and FSH, which are required for the development and maintenance of the gonads and thus for fertility, bind to receptors on the gonads to regulate gametogenesis and gonadal steroidogenesis in both sexes (Millar 2005; Muro et al., 2021).

Extensive research has shown that several neuropeptides have been viewed as modulators or regulators of GnRH neurons in the porcine hypothalamus, including Kisspeptins, which have a stimulating effect on the activity and synthesis of GnRH neurons, and RFamide-related peptide-3 (RFRP-3; gonadotropin inhibitory hormone, GnIH), which has an inhibitory effect on the activity and synthesis of GnRH neurons (Herbison 2016). It is now well established from a variety of studies, that the coordination effect of excitatory neuronal signaling coupled with inhibitor neuronal input to the GnRH pulse generator controls the function of the HPG axis, thereby driving and maintaining the reproductive ability of pigs (Spergel 2019). Although recent advancements in neuropeptides that regulate porcine GnRH have been made, our understanding of the central nervous system network of porcine reproduction remains incomplete. This review focuses on the neuropeptides which have been viewed as regulators of GnRH neuronal activity and/ or reproductive function and whether they act directly on GnRH neurons in pigs. The complete elucidation of the novel neuropeptidergic and molecular mechanisms summarized in this review will not only expand our knowledge of the intimate mechanisms responsible for the reproductive in pigs but might also provide new tools and targets for better prevention and management of pig reproduction in practice (Peltoniemi et al., 2019).

## The important role of gonadotropin-releasing hormone (GnRH) neurons in pig reproduction

The activation of the hypothalamic-pituitary axis is critical for the initiation and maintenance of reproductive cycles in pigs and is influenced by a number of factors, such as nutrition, metabolism, and gonadal steroids (Garcia et al., 2020; Marín-García and Llobat 2021). At present, it is universally admitted that GnRH neurons in pigs function as brain sensors and main effectors for the modulation of the hypothalamus level. The cell bodies of GnRH neurons, which receive neuropeptidergic inputs from neurons in the hypothalamus and other brain areas, are distributed in the preoptic area (POA) at the organum vasculosum of the lamina terminalis (OVLT) level, medial basal hypothalamus (MBH) including the arcuate nucleus (ARC), and in the anterior hypothalamic area (AHA) (Lents et al., 2020).

In pigs, GnRH pulsation is essential for maintaining gonadotropin gene expression and the physiological pattern of gonadotropin secretion. The pulse frequencies of GnRH and LH are known to change throughout the estrous cycle and postpartum period. In the luteal phase of gilts, a pattern of LH secretion characterized by high-amplitude, low-frequency pulses, and reduced serum concentrations of LH,as well as increased serum concentrations of FSH were associated with the low-frequency GnRH pulse. In the follicular phase of gilts, the transition of the GnRH pulse mode to a high frequency resulting in the pattern of LH secretion changing to high-frequency, low-amplitude pulses, and a decrease in FSH synthesis and release. The interaction between GnRH and LH/FSH found that the synthesis and pulsatile secretion of GnRH from neurons in the hypothalamus drives pulsatile secretion of LH, and to a lesser extent, FSH in gilts (Tsutsumi and Webster 2009).

## Neuropeptide modulators of GnRH neuronal activity, GnRH secretion and reproduction that act directly on GnRH neurons

### Kisspeptin

Kisspeptin is the peptide encoded by the KISS1 gene, and the Kisspeptin receptor is a G-protein-coupled receptor, GPR54 (Ohtaki et al., 2001; Seminara et al., 2003). Kisspeptin has emerged as a key regulator of reproductive function in pigs when it was discovered that boars in which a functional Kisspeptin receptor was knocked out with gene-editing technology presented a condition of hypogonadotropic hypogonadism (Sonstegard et al., 2017). The boars are characterized by a lack of gonadal development and low levels of gonadotropin secretion from the anterior pituitary gland that failed to transition through puberty (de Roux et al., 2003; Semple et al., 2005). A substantial body of evidence indicates that Kisspeptin has potent stimulatory action on the secretion of gonadotropin hormones in gilts (Lents et al., 2008; Ralph et al., 2017). Likewise, accumulating evidence showed that central and peripheral treatment with Kisspeptin stimulated gonadotropin secretion, particularly LH secretion, in various mammalian species, including rodents, sheep, goats, cattle, and horses (Thompson et al., 2004; Messager et al., 2005; Shahab et al., 2005; Caraty et al., 2007; Kadokawa et al., 2008; Magee et al., 2009; Hashizume et al., 2010). In sheep, Kisspeptin receptor is expressed in the GnRH neurons of the hypothalamus, and intracerebroventricular infusion of Kisspeptin caused a dramatic increase in serum LH and FSH,accompanied by a concomitant release of the cerebrospinal fluid GnRH content (Messager et al., 2005; Caraty et al., 2007; Smith et al., 2011). Moreover, Kisspeptin-induced LH secretion was abolished in ewes treated with neutralizing antibodies to GnRH, and in ewes in which the hypothalamus had been disconnected from the pituitary to eliminate GnRH input to gonadotroph cells, indicating that Kisspeptin stimulates LH secretion in a GnRH-dependent manner (Arreguin-Arevalo et al., 2007; Smith et al., 2008b). Although there are no similar studies in pigs, the direct action of Kisspeptin on GnRH neurons is also inferred from the spatial distribution of the Kisspeptin expression within the porcine hypothalamus.

The localization of the Kisspeptin expression within the porcine hypothalamus has not been fully characterized (Lents 2019). In the central nervous system of pigs, Kisspeptin cells are localized primarily in two discrete regions involved in the regulation of gonadotropin secretion, including the MBH within the ARC and the periventricular (PeV) nucleus (Tomikawa et al., 2010; Ieda et al., 2014; Thorson et al., 2017). Within the ARC of the pig, a spatially distinct pattern of KISS1 is seen, with the greatest expression occurring in the medio-caudal sections, similar to the ARC distribution of Kisspeptin observed in sheep and cattle (Redmond et al., 2011; Cardoso et al., 2015; Lents et al., 2020). Preliminary immunocytochemistry data illustrate that neuronal cell bodies as well as nerve fibers for Kisspeptin are evident in the porcine ARC. Thus, it is anticipated that the neuroanatomical distribution of Kisspeptin neurons in the porcine ARC is like that of other species. Specifically, Kisspeptin neurons in the POA regulate GnRH cell bodies, whereas Kisspeptin neurons in the ARC act on GnRH terminal axons in the median eminence (Lents et al., 2020).

Estradiol has a biphasic effect in pigs, inhibiting basal LH pulses via negative feedback then stimulating an ovulatory surge of LH through positive feedback (Ieda et al., 2014; Thorson et al., 2017). When sexually mature OVX gilts were given a dose of estradiol sufficient to stimulate an ovulatory surge of LH, the expression of Kisspeptin in the PeV was upregulated compared with control OVX gilts (Tomikawa et al., 2010; Silva et al., 2021). It is inferred that separate populations of Kisspeptin neurons in the ARC and the PeV of gilts mediate negative and positive estrogen feedback for the control of tonic and surge LH secretion, respectively. Moreover, previous research has established that the initiation of puberty and postpartum reproductive cycles in gilts are metabolically gated. Recent work by Thorson et al. (2018) has established that short-term (10 days) negative energy balance induced reduced frequency and increased amplitude of LH pulses, but no differences in ARC the transcription of Kisspeptin between feed-restricted and full-fed gilts were observed (Thorson et al., 2018). Surveys such as that conducted by Zhou et al. (2014) have shown that feed restriction to cyclic gilts for a prolonged period (100 days) resulted in the point that they ceased cycling, and mRNA expressions for Kisspeptin, Kisspeptin receptor, and GnRH were all downregulated in the MBH; on the contrary, Kisspeptin and its receptor mRNA expression were upregulated in the hypothalamic tissue containing the caudal POA and PeV of pigs fed a higher-energy diet (Zhou et al., 2014). This implies that nutrition-induced changes in LH pulse patterns of pigs may depend on hypothalamic subpopulations of Kisspeptin neurons that respond differently to nutritional signals in mediating the GnRH pulse generator.

### α-Melanocyte-stimulating hormone (α-MSH)

The anorexigenic neuropeptide α-MSH is synthesized and released by pro-opiomelanocortin (POMC) neurons and the α-MSH analogue melanotan II, which act directly via MC3Rs and MC4Rs on most (70%) GnRH neurons to increase their firing rate and induce the expression of c-Fos in rats (Israel et al., 2012; Roa and Herbison 2012; Lane and Whitaker 2018; Spergel 2019; Xu et al., 2021). In pigs, the distribution and location of POMC perikarya are identified specifically within and around the immediate location of the ARC. Fibers for POMC were noted as projecting rostrally from the ARC to the SCN, SOP, LHA, medial POA, and dbB (Lents et al., 2020). GnRH neurons in the hypothalamus of gilts showed numerous close contacts with POMC-containing varicosities, and both GnRH and POMC fibers have extensive overlap within the ME, supporting the expectation that α-MSH may control the release of GnRH and(or) Kisspeptin for increased LH pulses. Neither ICV treatment of prepubertal OVX gilts with a melanocortin agonist (NDP-MSH) or SHU9119 affected LH secretion, offering contradictory findings (Barb et al., 2004). Moreover, NPY expression increased, while promelanin-concentrating hormone expression decreased with no change in AGRP and POMC expression in NDP-MSH-treated pigs, suggesting α-MSH may is involved in regulating energy homeostasis to reproduction in pigs(Barb et al.,, 2010).

### RFamide-related peptide-3 (RFRP-3)

The RFamide-related peptide (RFRP) gene, an ortholog to the gonadotropin-inhibiting hormone (GnIH) gene that was first detected in the hypothalamic–hypophysial system of avian species and regulates avian reproduction by decreasing gonadotropin release and synthesis by acting on the GnRH system and the anterior pituitary gland, was found to encode 3 biological peptides, RFRP-1, RFRP-2, and RFRP-3 (Hinuma et al., 2000; Legagneux et al., 2009). The receptors of RFRP widely distributed in the central nervous system, pituitary, and gonads in pigs include GPR147 or NPFF1R, the canonical receptor for RFRP, and GPR74 or NPFF2R albeit with much less potency (Yoshida et al., 2003; Bentley et al., 2010). Compelling evidence has documented a role of RFRP-3 in regulating GnRH and GHRH neuronal expression and function as well as secretion of gonadotrophin and steroid hormones and impacting the expression of Kisspeptin in mammals (Johnson et al., 2007; Ducret et al., 2009; Kadokawa et al., 2009; Ancel et al., 2012; Li et al., 2013). The pig RFRP gene was found to be abundantly expressed in the nervous system (cerebellum, cerebrum, hypothalamus, and pituitary) and reproduction system (ovary, oviduct, uterus, and testis), and is thought to be a candidate gene for porcine reproductive traits (Fang et al., 2014).

Using in situ hybridization and immunohistochemistry, the spatial distribution of RFRP has been investigated in the porcine hypothalamus. RFRP-ir neuronal cell bodies and nerve fibers were located in the posterior hypothalamus, dorsomedial hypothalamic nucleus (DMH), and ventromedial hypothalamic nucleus (VMH), with a similar distribution as that in the brain of rodents, sheep, and mares (Yano et al., 2003; Kriegsfeld et al., 2006; Gibson et al., 2008; Smith et al., 2008a; Qi et al., 2009; Thorson et al., 2014). The most abundant population of porcine RFRP-ir was observed in the paraventricular nucleus of the hypothalamus (PVN), agreeing with observations in sheep and nonhuman primates (Yano et al., 2003; Kriegsfeld 2006; Smith et al., 2012). In sheep, RFRP fibers project from the PVN to the lateral hypothalamic area, the ARC, and the external zone of the ME, as well as being closely associated with many other neurons, such as GnRH, POMC, NPY, orexin, and Kisspeptin neurons (Smith et al., 2008a, 2012). Moderately dense RFRP fibers were observed in the lateral hypothalamic area of the porcine hypothalamus, but no RFRP fibers were observed in the external zone of the ME of gilts, which would suggest that RFRP is not released into the hypophyseal portal vasculature of the pig as has been observed in sheep (Li et al., 2013).

Early reports indicated that RFRP-3 attenuated GnRH-stimulated gonadotropin synthesis and the release from primary cultures of porcine anterior pituitary cells (Zmijewska et al., 2020). Furthermore, GnRH secretion from porcine hypothalamic explants was suppressed upon treatment with RFRP-3 (Li et al., 2013). Additionally, there was a study demonstrated that RFRP-3 inhibits the Kisspeptin-activated GnRH firing rate in vitro, but the addition of RFRP-3 to porcine cultured pituitary cells had no antagonistic effect on the Kisspeptin-induced stimulation of LH secretion (Wu et al., 2009; Zmijewska et al., 2020). These results indicate that RFRP-3 may be a negative regulator of pituitary gonadotropin synthesis and release via GnRH neurons. On the contrary, accumulating evidence further showed that central administration of RFRP-3 into the lateral ventricles of the brain within OVX gilts had no effect on the secretory patterns of LH (Thorson et al., 2017), which is consistent with the effects of RFRP-3 injected into the third ventricle of OVX ewes (Caraty et al., 2012; Decourt et al., 2016). When RFRP-3 was largely infused into the peripheral circulation of OVX gilts, LH secretion remained unchanged, unless very high doses were given. It also took large pharmacological doses of RFRP-3 to impact the pulsatile secretion of LH in intact mature boars (Thorson et al., 2015). As noted, the porcine RFRP preproprotein can yield an RFRP-2 peptide that ruminants and rodents do not produce (Yoshida et al., 2003). RFRP-2 is in the same position within the preproprotein as is avian GnIH and the amino acid sequence of porcine RFRP-2 has greater sequence homology with avian GnIH than RFRP-3, so it was speculated that RFRP-2 was a porcine-specific GnIH. However, when OVX gilts received infusions of RFRP-2 into the peripheral circulation, LH pulses were unaffected (Thorson et al., 2017). Because RFRP does not appear to have a potent suppressive effect on in vivo LH secretion in gilts, either centrally or peripherally, some authors have suggested that RFRP does not appear to act as a hypophysiotropic GnIH in pigs. Interestingly, the influence of RFRP on LH within mammals has been highly inconsistent; for instance, several studies have shown that RFRP-3 can inhibit, have no effect on, or even stimulate LH secretion (Yano et al., 2003; Murakami et al., 2008; Anderson et al., 2009; Rizwan et al., 2009; Pineda et al., 2010). It therefore remains unclear what role RFRP plays in porcine reproduction, requiring further research and analysis for pigs.

### Endogenous opioid peptides (EOP)

Endogenous opioid peptides (EOP) consist collectively of endorphins, enkephalins, and dynorphins, playing important roles in suppressing LH secretion in luteal phase gilts and lactating sows. Immunocytochemical studies in gilts demonstrated the existence of pro-opiomelanocortin (POMC) perikarya in the ARC with fibers projecting to the MBH, PeV, ME, and preoptic area (Weems et al., 2018). Following intracerebroventricular (ICV) treatment with morphine, an EOP agonist, decreased secretion of LH, and FSH in prepubertal OVX gilts. In contrast, the general opioid receptor antagonist, naloxone, increased LH release in prepubertal gilts. Naloxone stimulated GnRH secretion from the hypothalamic-preoptic area collected from gilts, indicating that the EOP may inhibit LH secretion by action at the central nervous system. Moreover, naloxone failed to increase LH release in boars given antisera against GnRH. An adenohypophysial site of action in modulating LH release is supported by in vitro studies in which β-endorphin decreased basal and GnRH-induced LH secretion by porcine pituitary cells. In contrast, exposure to naloxone increased basal secretion of LH and enhanced pituitary responsiveness to GnRH (Wylot et al., 2013).

### Neuropeptide Y (NPY)

Neuropeptide Y (NPY) is a 36-amino-acid tyrosine-rich peptide that was first isolated from porcine brain extracts in 1982 and belongs to the “NPY family” of biologically active peptides. It includes two gut hormones: peptide YY (PYY) and pancreatic polypeptide (PP). NPY affects target cells by activating various G-coupled receptors belonging to the rhodopsin-like superfamily of receptors (Siawrys and Buchowski, 2018). Additionally, NPY as an orexigenic peptide is simultaneously involved in the modulation of the GnRH/LH system and could provide a link between nutrition and reproduction. In the pig, NPY receptors are widely distributed in the structures of the central nervous system and innervating peripheral organs, with a predominant localization in stalk-median eminence (SME), POA, MBH, and the pituitary (Óvilo et al., 2010). NPY is widespread in the porcine CNS, including the limbic system, olfactory system, hypothalamoneurohypophysial tract, corpus striatum, and cerebral cortex. Barb revealed that the central administration of NPY suppresses serum LH concentrations and LH pulse frequency in OVX prepubertal gilts (Barb et al., 2006). Generally, based on studies performed with different species, there are many reports indicating that NPY may regulate LH secretion at the hypothalamic level by directly and/or indirectly modulating the activity of the GnRH neuronal system (Wójcik-Gładysz and Polkowska 2006; Dhillon et al., 2009; Roa and Herbison 2012; Amstalden et al., 2014). Moreover, morphological studies have proved that NPY neurons come in close contact with GnRH neurons in POA, ARC, and ME (Klenke et al., 2010). It is expected that NPY suppresses LH pulse frequency by inhibiting both GnRH and Kisspeptin cells in the gilt hypothalamus, but this problem requires further and more detailed investigation, including on the use of specific agonists and/or antagonists of all NPY receptor subtypes.

### Galanin (GAL)

Galanin (GAL) is a brain-gut neuropeptide widely distributed in the POA, MBH, and pituitary stalk-median eminence of the cyclic gilt (Czujkowska and Arciszewski 2016). The anorexigenic neuropeptide GAL expression and immunoreactivity are regulated by Estrogen (Spergel 2019). GAL is likely co-released with Kisspeptin from subsets of Kisspeptin neuron axons onto GnRH neurons and both the GALR1 and GALR2 subtypes of GAL receptor appear to be expressed in GnRH neurons (Constantin and Wray 2016). GAL stimulated basal but not GnRH-induced LH secretion from porcine pituitary glands in vitro and antiserum to galanin suppressed GnRH-induced LH release (Elsaesser 2001).

## Neuropeptides that may or may not act directly on GnRH neurons

### Neurokinin B (NKB)

Inactivating mutations in genes encoding neurokinin B (NKB) or its receptor NK3R results in hypogonadotropic hypogonadism and failure to attain puberty in rodents and humans, a phenotype reminiscent of that of patients with mutations of Kiss1 or GPR54 (de Roux et al., 2003; Topaloglu et al., 2009). In addition, initial studies in mice and sheep, later confirmed in other species, found that the Kisspeptin neurons in the ARC co-express neurokinin B (NKB), also known as tachykinin 3 (TAC3), which acts through its homologous receptor (TAC3R) (Burke et al., 2006; Goodman et al., 2007; Navarro et al., 2009). Similarly, studies in pig ARC have shown that virtually all Kisspeptin neurons in co-express NKB (Lents et al., 2020). Because the roles of NKB and its receptor in regulating GnRH of the pig are not presently understood, the expression of TAC3R has recently been localized throughout several areas of the hypothalamus in ovariectomized (OVX) gilts (Lindo 2018). Many of the hypothalamic regions that display TAC3R immunostaining in the pig also contain GnRH neurons. Furthermore, the greatest number of TAC3R expressing cells are the dbB and the PVN, followed by the POA and SCN. The PeV and RCh have evidence of TAC3R immunostaining (Lindo 2018). Although about 40% of GnRH neurons occurred in close apposition to TAC3R containing cells, close contacts were few, suggesting that the TAC3R containing cells in the porcine hypothalamus do not directly regulate GnRH neurons in pigs.

It is postulated from molecular, anatomical, and physiological data that NKB acts as an autoregulatory signal for Kisspeptin-neurokin B-dynorphin (KNDy) neurons, stimulating Kisspeptin output to GnRH neurons. The fact that NKB operates via Kisspeptin signaling to modulate GnRH neurons is supported by a wealth of data, including the demonstration that within monkeys the desensitization of TAC3R blocks the effect of senktide (an NKB agonist) on gonadotropin release, and the fact that ICV injection of senktide into rodents induces c-Fos in ARC Kiss1 neurons, which express NK3R and are excited by NKB (Navarro et al., 2009, 2011; Ramaswamy et al., 2011). Moreover, the effect of senktide is absent in GPR54 null mice but is preserved in mice engineered to maintain Kisspeptin actions only in GnRH neurons, attesting that NKB signaling is upstream of Kisspeptin in the control of GnRH neurons (de Croft et al., 2013). There is little published data on NKB in pigs, but the spatial distribution of NKB expression within the porcine hypothalamus has inferred the function of NKB on GnRH neurons as well. Furthermore, some studies in the gilt suggested that the amplitude of LH pulses may be regulated by NKB, and the pubertal decrease in sensitivity to oestrogen negative feedback in the gilt involves the gene expression for TAC3 and the TAC3R gene in the medial basal hypothalamus (MBH) (Thorson et al., 2018). Thus, it is likely that NKB in pigs acts through an autoregulatory mechanism involving TAC3R to induce the release of Kisspeptin, which acts directly on the GnRH neuronal network to stimulate a pulsatile release of GnRH and subsequently LH.

### Neuromedin B (NMB)

Neuromedin B (NMB) is a member of a family of bombesin-like peptides in mammals, which are decapeptides originally identified in porcine spinal cords (Jensen et al., 2008). Amino acid sequences from sequenced cDNA show that the Neuromedin B receptor (NMBR) in pigs is typical of the G protein-coupled receptor (GPCR) family, serving as a 390-amino acid protein with seven membrane spanning domains (Ohki-Hamazaki et al., 2005). NMB/NMBR is an important physiological regulator of smooth muscle contraction, via activation of intracellular signaling pathways. The expression of NMB mRNA in the central nervous system (CNS) as well the presence of NMBR mRNA and protein in the pituitary, testis, ovaries, and uterus, suggest the potential physiological functions of the NMB/NMBR system during reproduction in pigs (Ma et al., 2016). Moreover, the expression patterns of NMB and NMBR mRNA along the reproductive axis for female pigs across the estrous cycle and for male pigs at postnatal development stages, support the suggestion that NMB may control the release of GnRH through the regulation of NMBR secretion (Ma et al., 2018). It has previously been shown that NMB can stimulate the HPG axis via hypothalamic GnRH in male rats (Boughton et al., 2013). However, the mechanism and physiological function of the NMB/NMBR system on porcine reproduction are currently not fully understood, requiring further research and analysis.

### Neuromedin S (NMS)

Neuromedin S (NMS) has been reported to have many physiological functions in mammals and has been identified as an endogenous ligand for two orphan G protein-coupled receptors, FM-3/GPR66 (NMU1R) and FM-4/TGR-1 (NMU2R)(Roesler et al., 2012). The distribution and location of NMS have mainly been identified in the pig’s hypothalamic region, including in the periventricular nucleus (PEN), PVN, SCN, supraoptic nucleus (SON), VMH, and ARC (Mori et al., 2005). NMU2R is widely distributed in male pig hypothalamic cells, anterior pituitary cells, and Leydig cells, suggesting that the NMS/NMU2R system existed in the male pig reproductive axis and may play a significant role in the regulation of gonadotropin secretion in the brain and testis, which was similar to its function in other mammals (Yang et al., 2012; Ma et al., 2017). At present, evidence has revealed that NMS increases the release of LH and FSH from anterior pituitary cells and testosterone from Leydig cells as well as the expression of NMU2R and GnRH mRNAs in hypothalamic cells, NMU2R, LH, and FSH mRNAs in anterior pituitary cells; moreover, it downregulated the expression of GnIH mRNA in hypothalamic cells (Jin et al., 2019). Interestingly, lateral ventricle injection of NMS could significantly decrease LH response including serum LH level and LH mRNA expression in ovariectomized pigs, which is partially consistent with the previous results on rats (Yang et al., 2010). The inhibitory effect of NMS on LH contradicts previous reports of a positive role of NMS on the reproductive axis in vitro, but these results indicated that NMS may play an important role in the regulation of reproductive function via the NMU2R or GnRH.

### Nesfatin-1

The hypothalamic peptide, Nesfatin-1, derived from the precursor NEFA/nucleobindin 2 (NUCB2), was identified as anorexigenic signal, acting in a leptin-independent manner (Garcia-Galiano et al., 2010). Nesfatin-1 is expressed in the ventrolateral medulla (VLM), dorsal vagal complex (DVC), PVN, ARC, and SON of the pig brain (Gaigé et al., 2013). Accumulating evidence indicated that Nesfatin-1 regulates glucose metabolism, insulin secretion, gastrointestinal motility, stress response, development, cardiovascular functions, anxiety, and the onset of puberty (Aydin, 2013). Nesfatin-1 stimulates the secretion of GnRH and LHβ in vitro, raising the possibility of Nesfatin-1 acting directly on hypothalamic neurons and gonadotropes (Kalló et al., 2012).

### Phoenixin (PNX)

Phoenixin (PNX) is a recently discovered hypothalamic neuropeptide, first identified in 2013 (Yosten et al., 2013). Phoenixin acts through its receptor, G protein-coupled receptor 173 (GPR173), to activate the cAMP/PKA pathway resulting in the phosphorylation of CREB (pCREB) (Treen et al., 2016). PXN potentiates GnRH-stimulated LH release and increases GnRH and KISS1 gene expression, respectively, and it also raises the expression of GnRH receptor gene (Clarke and Dhillo, 2019). Alternatively, compromise of PXN in vivo using siRNA led to a reduction in GnRH receptor expression in the pituitary and the delayed appearance of oestrus (Nguyen et al., 2019). Until now, it is expected that PNX may have important roles in the regulation of porcine reproductive function, yet to be delineated (Lepiarczyk et al., 2020).

## Conclusion

**Figure 1:**
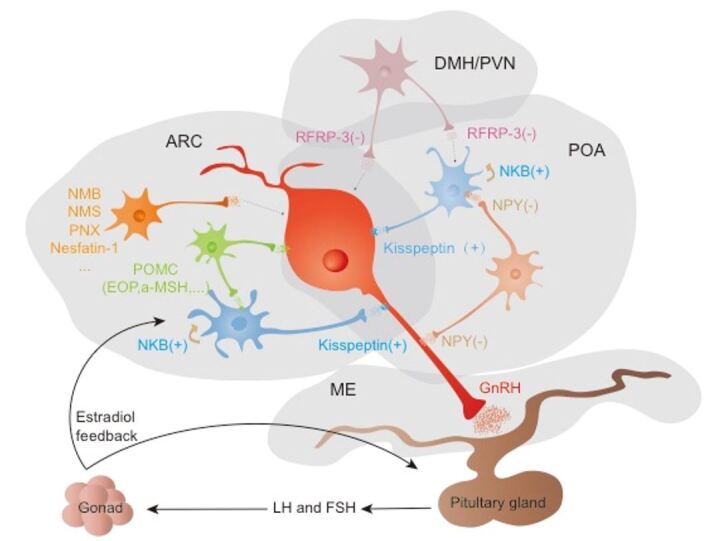
Schematic illustration of the reproductive neuroendocrine pathways in pigs. The reproduction of pigs is operated by the HPG axis, in which GnRH neurons act as the intermediate factor between the hypothalamus and hypophysis. GnRH neurons directly or indirectly receive regulatory inputs from a wide array of regulatory signals and pathways, involving numerous neuropeptides and neurotransmitters. Kisspeptin neurons with neurokinin B in the POA regulate GnRH cell bodies and in the ARC act on GnRH terminal axons in the median eminence, stimulating GnRH secretion. NPY and POMC cells function as metabolic sensors for the activation of GnRH secretion, acting as inhibitory and excitatory signals, respectively. Rather, it is speculated that RFRP-3, NMB, NMS, PNX, GAL, and Nesfatin-1 act as the essential upstream regulators in the control of GnRH secretion, but remains unclear.

In this review, beginning from the anatomic distribution and pharmacological function of neuropeptides, we follow the neuroendocrine pathways and mechanisms that control reproduction in pigs. GnRH neurons play a particularly critical role in the function of the reproductive central nervous system and act as the intermediate factor between the hypothalamus and hypophysis. The activity of GnRH neurons is regulated by different neuropeptides, forming a central control network. Kisspeptin neurons along with neurokinin B in the POA regulate GnRH cell bodies and in the ARC act on GnRH terminal axons in the median eminence, which is essential for GnRH neurons to stimulate LH secretion in pigs. Rather, it is speculated that RFRPs act as the essential upstream regulators in the control of GnRH secretion with an inhibitory effect in pigs, as has been proposed for rats and nonhuman primates, but remains unclear. NPY and POMC cells function as metabolic sensors for the activation of GnRH secretion, acting as inhibitory and excitatory signals, respectively. Additionally, EOP inhibition of GnRH secretion in pigs involves the direct suppression of noradrenergic neurons, which may come about with increasing sexual maturity. Several neuropeptides may play an important role in the regulation of reproductive functions via hypothalamic GnRH, such as NMB, NMS, PNX, GAL, Nesfatin-1, and as such, require further research and analysis (Figure 1).

The list of neuropeptides known to modulate or potentially modulate GnRH neuronal activity and GnRH secretion in gilts will likely grow as the effects on GnRH neurons of additional neuropeptides, including those of neuropeptides that are yet to be discovered, are investigated. Although it is clear, based mostly on pharmacological and immunohistochemical studies, that some of the neuropeptides investigated thus far impact directly GnRH neurons and the receptors to which they bind on GnRH neurons, further research is required to understand their signaling mechanisms in GnRH neurons and to determine whether other known and yet undiscovered neuropeptides affect GnRH neurons directly or indirectly.

## References

[B001] Amstalden M, Cardoso RC, Alves BRC, Williams GL (2014). Reproduction Symposium: hypothalamic neuropeptides and the nutritional programming of puberty in heifers1,2. J Anim Sci.

[B002] Ancel C, Bentsen AH, Sébert M-E, Tena-Sempere M, Mikkelsen JD, Simonneaux V (2012). Stimulatory effect of RFRP-3 on the gonadotrophic axis in the male syrian hamster: the exception proves the rule. Endocrinology.

[B003] Anderson GM, Relf H-L, Rizwan MZ, Evans JJ (2009). Central and peripheral effects of RFamide-Related Peptide-3 on luteinizing hormone and prolactin secretion in rats. Endocrinology.

[B004] Arreguin-Arevalo JA, Lents CA, Farmerie TA, Nett TM, Clay CM (2007). KiSS-1 peptide induces release of LH by a direct effect on the hypothalamus of ovariectomized ewes. Anim Reprod Sci.

[B005] Aydin S (2013). Multi-functional peptide hormone NUCB2/nesfatin-1. Endocrine.

[B006] Barb CR, Robertson AS, Barrett JB, Kraeling RR, Houseknecht KL (2004). The role of melanocortin-3 and -4 receptor in regulating appetite, energy homeostasis and neuroendocrine function in the pig. J Endocrinol.

[B007] Barb CR, Kraeling RR, Rampacek GB, Hausman GJ (2006). The role of neuropeptide Y and interaction with leptin in regulating feed intake and luteinizing hormone and growth hormone secretion in the pig. Reproduction.

[B008] Barb CR, Hausman GJ, Rekaya R, Lents CA, Lkhagvadorj S, Qu L, Cai W, Couture OP, Anderson LL, Dekkers JC, Tuggle CK (2010). Gene expression in hypothalamus, liver, and adipose tissues and food intake response to melanocortin-4 receptor agonist in pigs expressing melanocortin-4 receptor mutations. Physiol Genomics.

[B009] Bentley GE, Tsutsui K, Kriegsfeld LJ (2010). Recent studies of gonadotropin-inhibitory hormone (GnIH) in the mammalian hypothalamus, pituitary and gonads. Brain Res.

[B010] Boughton CK, Patel SA, Thompson EL, Patterson M, Curtis AE, Amin A, Chen K, Ghatei MA, Bloom SR, Murphy KG (2013). Neuromedin B stimulates the hypothalamic–pituitary–gonadal axis in male rats. Regul Pept.

[B011] Burke MC, Letts PA, Krajewski SJ, Rance NE (2006). Coexpression of dynorphin and neurokinin B immunoreactivity in the rat hypothalamus: morphologic evidence of interrelated function within the arcuate nucleus. J Comp Neurol.

[B012] Caraty A, Smith JT, Lomet D, Ben Saïd S, Morrissey A, Cognie J, Doughton B, Baril G, Briant C, Clarke IJ (2007). Kisspeptin Synchronizes Preovulatory Surges in Cyclical Ewes and Causes Ovulation in Seasonally Acyclic Ewes. Endocrinology.

[B013] Caraty A, Blomenröhr M, Vogel GMT, Lomet D, Briant C, Beltramo M (2012). RF9 powerfully stimulates gonadotrophin secretion in the Ewe: evidence for a seasonal threshold of sensitivity. J Neuroendocrinol.

[B014] Cardoso RC, Alves BRC, Sharpton SM, Williams GL, Amstalden M (2015). Nutritional programming of accelerated puberty in heifers: involvement of pro‐opiomelanocortin neurones in the arcuate nucleus. J Neuroendocrinol.

[B015] Clarke SA, Dhillo WS (2019). Phoenixin and its role in reproductive hormone release. Semin Reprod Med.

[B016] Constantin S, Wray S (2016). Galanin activates G protein gated inwardly rectifying potassium channels and suppresses Kisspeptin-10 activation of GnRH neurons. Endocrinology.

[B017] Czujkowska A, Arciszewski MB (2016). Galanin is Co-Expressed with Substance P, Calbindin and Corticotropin-Releasing Factor (CRF) in The Enteric Nervous System of the Wild Boar (*Sus scrofa*) Small Intestine. Anat Histol Embryol.

[B018] de Croft S, Boehm U, Herbison AE (2013). Activates arcuate kisspeptin neurons through multiple tachykinin receptors in the male mouse. Endocrinology.

[B019] de Roux N, Genin E, Carel J-C, Matsuda F, Chaussain J-L, Milgrom E (2003). Hypogonadotropic hypogonadism due to loss of function of the KiSS1-derived peptide receptor GPR54. Proc Natl Acad Sci USA.

[B020] Decourt C, Anger K, Robert V, Lomet D, Bartzen-Sprauer J, Caraty A, Dufourny L, Anderson G, Beltramo M (2016). No Evidence That RFamide-Related Peptide 3 Directly Modulates LH Secretion in the Ewe. Endocrinology.

[B021] Dhillon SS, Gingerich S, Belsham DD (2009). Neuropeptide Y induces gonadotropin-releasing hormone gene expression directly and through conditioned medium from mHypoE-38 NPY neurons. Regul Pept.

[B022] Ducret E, Anderson GM, Herbison AE (2009). RFamide-related peptide-3, a mammalian gonadotropin-inhibitory hormone ortholog, regulates gonadotropin-releasing hormone neuron firing in the mouse. Endocrinology.

[B023] Elsaesser F (2001). Stimulation of porcine pituitary luteinizing hormone release by galanin: putative auto/paracrine regulation. Neuroendocrinology.

[B024] Fang MX, Huang YS, Ye J, Zhang W, Li Y, Nie QH (2014). Identification and characterization of RFRP gene in pigs and its association with reproductive traits. Genet Mol Res.

[B025] Gaigé S, Bonnet MS, Tardivel C, Pinton P, Trouslard J, Jean A, Guzylack L, Troadec JD, Dallaporta M (2013). c-Fos immunoreactivity in the pig brain following deoxynivalenol intoxication: focus on NUCB2/nesfatin-1 expressing neurons. Neurotoxicology.

[B026] Garcia IS, Teixeira SA, Costa KA, Marques DBD, Rodrigues GA, Costa TC, Guimarães JD, Otto PI, Saraiva A, Ibelli AMG, Cantão ME, Oliveira HC, Ledur MC, Peixoto JO, Guimarães SEF (2020). L‐Arginine supplementation of gilts during early gestation modulates energy sensitive pathways in pig conceptuses. Mol Reprod Dev.

[B027] Garcia-Galiano D, Navarro VM, Roa J, Ruiz-Pino F, Sanchez-Garrido MA, Pineda R, Castellano JM, Romero M, Aguilar E, Gaytán F, Diéguez C, Pinilla L, Tena-Sempere M (2010). The anorexigenic neuropeptide, Nesfatin-1, Is indispensable for normal puberty onset in the female rat. J Neurosci.

[B028] Gibson EM, Humber SA, Jain S, Williams WP, Zhao S, Bentley GE, Tsutsui K, Kriegsfeld LJ (2008). Alterations in RFamide-related peptide expression are coordinated with the preovulatory luteinizing hormone surge. Endocrinology.

[B029] Goodman RL, Lehman MN, Smith JT, Coolen LM, de Oliveira CVR, Jafarzadehshirazi MR, Pereira A, Iqbal J, Caraty A, Ciofi P, Clarke IJ (2007). Kisspeptin neurons in the arcuate nucleus of the ewe express both dynorphin A and neurokinin B. Endocrinology.

[B030] Hashizume T, Saito H, Sawada T, Yaegashi T, Ezzat AA, Sawai K, Yamashita T (2010). Characteristics of stimulation of gonadotropin secretion by kisspeptin-10 in female goats. Anim Reprod Sci.

[B031] Herbison AE (2016). Control of puberty onset and fertility by gonadotropin-releasing hormone neurons. Nat Rev Endocrinol.

[B032] Hinuma S, Shintani Y, Fukusumi S, Iijima N, Matsumoto Y, Hosoya M, Fujii R, Watanabe T, Kikuchi K, Terao Y, Yano T, Yamamoto T, Kawamata Y, Habata Y, Asada M, Kitada C, Kurokawa T, Onda H, Nishimura O, Tanaka M, Ibata Y, Fujino M (2000). New neuropeptides containing carboxy-terminal RFamide and their receptor in mammals. Nat Cell Biol.

[B033] Ieda N, Uenoyama Y, Tajima Y, Nakata T, Kano M, Naniwa Y, Watanabe Y, Minabe S, Tomikawa J, Inoue N, Matsuda F, Ohkura S, Maeda K, Tsukamura H (2014). KISS1 Gene Expression in the Developing Brain of Female Pigs in Pre- and Peripubertal Periods. J Reprod Dev.

[B034] Israel DD, Sheffer-Babila S, de Luca C, Jo Y-H, Liu SM, Xia Q, Spergel DJ, Dun SL, Dun NJ, Chua SC (2012). Effects of Leptin and Melanocortin Signaling Interactions on Pubertal Development and Reproduction. Endocrinology.

[B035] Jensen RT, Battey JF, Spindel ER, Benya RV (2008). International Union of Pharmacology. LXVIII. Mammalian Bombesin Receptors: Nomenclature, Distribution, Pharmacology, Signaling, and Functions in Normal and Disease States. Pharmacol Rev.

[B036] Jin M, Ma Z, Li X, Su J, Lei Z (2019). The effects of neuromedin S on the hypothalamic-pituitary-testicular axis in male pigs in vitro. Gen Comp Endocrinol.

[B037] Johnson MA, Tsutsui K, Fraley GS (2007). Rat RFamide-related peptide-3 stimulates GH secretion, inhibits LH secretion, and has variable effects on sex behavior in the adult male rat. Horm Behav.

[B038] Kadokawa H, Matsui M, Hayashi K, Matsunaga N, Kawashima C, Shimizu T, Kida K, Miyamoto A (2008). Peripheral administration of kisspeptin-10 increases plasma concentrations of GH as well as LH in prepubertal Holstein heifers. J Endocrinol.

[B039] Kadokawa H, Shibata M, Tanaka Y, Kojima T, Matsumoto K, Oshima K, Yamamoto N (2009). Bovine C-terminal octapeptide of RFamide-related peptide-3 suppresses luteinizing hormone (LH) secretion from the pituitary as well as pulsatile LH secretion in bovines. Domest Anim Endocrinol.

[B040] Kalló I, Vida B, Deli L, Molnár CS, Hrabovszky E, Caraty A, Ciofi P, Coen CW, Liposits Z (2012). Co-localisation of kisspeptin with galanin or neurokinin B in afferents to mouse GnRH neurones. J Neuroendocrinol.

[B041] Klenke U, Constantin S, Wray S (2010). Directly Inhibits neuronal activity in a subpopulation of gonadotropin-releasing hormone-1 neurons via Y1 receptors. Endocrinology.

[B042] Kriegsfeld LJ, Mei DF, Bentley GE, Ubuka T, Mason AO, Inoue K, Ukena K, Tsutsui K, Silver R (2006). Identification and characterization of a gonadotropin-inhibitory system in the brains of mammals. Proc Natl Acad Sci USA.

[B043] Kriegsfeld LJ (2006). Driving reproduction: RFamide peptides behind the wheel. Horm Behav.

[B044] Lane RL, Whitaker BD (2018). Melatonin and tannic acid supplementation in vitro improve fertilization and embryonic development in pigs. Anim Reprod.

[B045] Legagneux K, Bernard-Franchi G, Poncet F, La Roche A, Colard C, Fellmann D, Pralong F, Risold PY (2009). Distribution and genesis of the RFRP-producing neurons in the rat brain: comparison with melanin-concentrating hormone- and hypocretin-containing neurons. Neuropeptides.

[B046] Lents CA, Heidorn NL, Barb CR, Ford JJ (2008). Central and peripheral administration of kisspeptin activates gonadotropin but not somatotropin secretion in prepubertal gilts. Reproduction.

[B047] Lents CA, Lindo AN, Hileman SM, Nonneman DJ (2020). Physiological and genomic insight into neuroendocrine regulation of puberty in gilts. Domest Anim Endocrinol.

[B048] Lents CA (2019). Review: kisspeptin and reproduction in the pig. Animal.

[B049] Lepiarczyk E, Bossowska A, Majewska M, Skowrońska A, Kaleczyc J, Majewski M (2020). Distribution and chemical coding of phoenixin-immunoreactive nerve structures in the spinal cord of the pig. Ann Anat.

[B050] Li X, Su J, Fang R, Zheng L, Lei R, Wang X, Lei Z, Jin M, Jiao Y, Hou Y, Guo T, Ma Z (2013). The effects of RFRP-3, the mammalian ortholog of GnIH, on the female pig reproductive axis in vitro. Mol Cell Endocrinol.

[B051] Lindo A (2018). Characterization of KNDy neuronal activity in gilts: distribution and effect of a progestin.

[B052] Ma Z, Su J, Guo T, Jin M, Li X, Lei Z, Hou Y, Li X, Jia C, Zhang Z, Ahmed E (2016). Neuromedin B and its receptor: gene cloning, tissue distribution and expression levels of the reproductive axis in pigs. PLoS ONE.

[B053] Ma Z, Zhao Y, Yao Y, Lei Z, Jin M, Li X, Jia C, Zhang Z, Li X, Su J (2017). Postnatal developmental of Neuromedin S and its receptor in the male Xiaomeishan pig reproductive axis. Anim Reprod Sci.

[B054] Ma Z, Zhang Y, Su J, Yang S, Qiao W, Li X, Lei Z, Cheng L, An N, Wang W, Feng Y, Zhang J (2018). Effects of neuromedin B on steroidogenesis, cell proliferation and apoptosis in porcine Leydig cells. J Mol Endocrinol.

[B055] Magee C, Foradori CD, Bruemmer JE, Arreguin-Arevalo JA, McCue PM, Handa RJ, Squires EL, Clay CM (2009). Biological and anatomical evidence for kisspeptin regulation of the hypothalamic-pituitary-gonadal axis of estrous horse mares. Endocrinology.

[B056] Marín-García PJ, Llobat L (2021). How does protein nutrition affect the epigenetic changes in pig? A review. Animals (Basel).

[B057] Messager S, Chatzidaki EE, Ma D, Hendrick AG, Zahn D, Dixon J, Thresher RR, Malinge I, Lomet D, Carlton MB, Colledge WH, Caraty A, Aparicio SA (2005). Kisspeptin directly stimulates gonadotropin-releasing hormone release via G protein-coupled receptor 54. Proc Natl Acad Sci USA.

[B058] Millar RP (2005). GnRHs and GnRH receptors. Anim Reprod Sci.

[B059] Mori K, Miyazato M, Ida T, Murakami N, Serino R, Ueta Y, Kojima M, Kangawa K (2005). Identification of neuromedin S and its possible role in the mammalian circadian oscillator system. EMBO J.

[B060] Murakami M, Matsuzaki T, Iwasa T, Yasui T, Irahara M, Osugi T, Tsutsui K (2008). Hypophysiotropic role of RFamide-related peptide-3 in the inhibition of LH secretion in female rats. J Endocrinol.

[B061] Muro BBD, Leal DF, Carnevale RF, Torres MA, Mendonça MV, Nakasone DH, Martinez CHG, Ravagnani GM, Monteiro MS, Poor AP, Martins SMMK, Viau P, Oliveira CA, Castro RVG, Bessi BW, Bressan FF, Pulz LH, Strefezzi RF, Almond GW, Andrade AFC (2021). Altrenogest during early pregnancy modulates uterine glandular epithelium and endometrial growth factor expression at the time implantation in pigs. Anim Reprod.

[B062] Navarro VM, Castellano JM, McConkey SM, Pineda R, Ruiz-Pino F, Pinilla L, Clifton DK, Tena-Sempere M, Steiner RA (2011). Interactions between kisspeptin and neurokinin B in the control of GnRH secretion in the female rat. Am J Physiol Endocrinol Metab.

[B063] Navarro VM, Gottsch ML, Chavkin C, Okamura H, Clifton DK, Steiner RA (2009). Regulation of Gonadotropin-Releasing Hormone Secretion by Kisspeptin/Dynorphin/Neurokinin B Neurons in the Arcuate Nucleus of the Mouse. J Neurosci.

[B064] Nguyen XP, Nakamura T, Osuka S, Bayasula B, Nakanishi N, Kasahara Y, Muraoka A, Hayashi S, Nagai T, Murase T, Goto M, Iwase A, Kikkawa F (2019). Effect of the neuropeptide phoenixin and its receptor GPR173 during folliculogenesis. Reproduction.

[B065] Ohki-Hamazaki H, Iwabuchi M, Maekawa F (2005). Development and function of bombesin-like peptides and their receptors. Int J Dev Biol.

[B066] Ohtaki T, Shintani Y, Honda S, Matsumoto H, Hori A, Kanehashi K, Terao Y, Kumano S, Takatsu Y, Masuda Y, Ishibashi Y, Watanabe T, Asada M, Yamada T, Suenaga M, Kitada C, Usuki S, Kurokawa T, Onda H, Nishimura O, Fujino M (2001). Metastasis suppressor gene KiSS-1 encodes peptide ligand of a G-protein-coupled receptor. Nature.

[B067] Óvilo C, Fernández A, Fernández AI, Folch JM, Varona L, Benítez R, Nuñez Y, Rodríguez C, Silió L (2010). Hypothalamic expression of porcine leptin receptor (LEPR), neuropeptide Y (NPY), and cocaine- and amphetamine-regulated transcript (CART) genes is influenced by LEPR genotype. Mamm Genome.

[B068] Peltoniemi O, Björkman S, Oropeza-Moe M, Oliviero C (2019). Developments of reproductive management and biotechnology in the pig. Anim Reprod.

[B069] Pineda R, Garcia-Galiano D, Sanchez-Garrido MA, Romero M, Ruiz-Pino F, Aguilar E, Dijcks FA, Blomenröhr M, Pinilla L, van Noort PI, Tena-Sempere M (2010). Characterization of the inhibitory roles of RFRP3, the mammalian ortholog of GnIH, in the control of gonadotropin secretion in the rat: in vivo and in vitro studies. Am J Physiol Endocrinol Metab.

[B070] Qi Y, Oldfield BJ, Clarke IJ (2009). Projections of RFamide-related peptide-3 neurones in the ovine hypothalamus, with special reference to regions regulating energy balance and reproduction. J Neuroendocrinol.

[B071] Ralph C, Kirkwood R, Tilbrook A (2017). A single intravenous injection of Kisspeptin evokes an increase in luteinising hormone in 15- and 18- week-old gilts. Anim Reprod Sci.

[B072] Ramaswamy S, Seminara SB, Plant TM (2011). Evidence from the agonadal juvenile male rhesus monkey (Macaca mulatta) for the view that the action of neurokinin B to Trigger Gonadotropin-Releasing Hormone Release Is Upstream from the Kisspeptin Receptor. Neuroendocrinology.

[B073] Redmond JS, Baez-Sandoval GM, Spell KM, Spencer TE, Lents CA, Williams GL, Amstalden M (2011). Developmental Changes in Hypothalamic Kiss1 Expression during Activation of the Pulsatile Release of Luteinising Hormone in Maturing Ewe Lambs. J Neuroendocrinol.

[B074] Rizwan MZ, Porteous R, Herbison AE, Anderson GM (2009). Cells expressing RFamide-related peptide-1/3, the mammalian gonadotropin-inhibitory hormone orthologs, are not hypophysiotropic neuroendocrine neurons in the rat. Endocrinology.

[B075] Roa J, Herbison AE (2012). Direct regulation of gnrh neuron excitability by arcuate nucleus POMC and NPY neuron neuropeptides in female mice. Endocrinology.

[B076] Roesler R, Kent P, Schröder N, Schwartsmann G, Merali Z (2012). Bombesin receptor regulation of emotional memory. Rev Neurosci.

[B077] Seminara SB, Messager S, Chatzidaki EE, Thresher RR, Acierno JS, Shagoury JK, Bo-Abbas Y, Kuohung W, Schwinof KM, Hendrick AG, Zahn D, Dixon J, Kaiser UB, Slaugenhaupt SA, Gusella JF, O’Rahilly S, Carlton MBL, Crowley WF, Aparicio SAJR, Colledge WH (2003). The GPR54 gene as a regulator of puberty. N Engl J Med.

[B078] Semple RK, Achermann JC, Ellery J, Farooqi IS, Karet FE, Stanhope RG, O’rahilly S, Aparicio SA (2005). Two novel missense mutations in G protein-coupled receptor 54 in a patient with hypogonadotropic hypogonadism. J Clin Endocrinol Metab.

[B079] Shahab M, Mastronardi C, Seminara SB, Crowley WF, Ojeda SR, Plant TM (2005). Increased hypothalamic GPR54 signaling: A potential mechanism for initiation of puberty in primates. Proc Natl Acad Sci USA.

[B080] Siawrys G, Buchowski H (2018). Modulation of anterior pituitary cell luteinizing hormone secretory activity by neuropeptide Y in early pregnant pigs. J Physiol Pharmacol.

[B081] Silva PCP, Brasil OO, Souto PLG, Moreira NH, Silva JP, Silva BDM, Ramos AF (2021). Fixed-time artificial insemination protocols on brazilian locally adapted breed gilts on ovulatory response and embryo production. Anim Reprod.

[B082] Smith JT, Coolen LM, Kriegsfeld LJ, Sari IP, Jaafarzadehshirazi MR, Maltby M, Bateman K, Goodman RL, Tilbrook AJ, Ubuka T, Bentley GE, Clarke IJ, Lehman MN (2008). Variation in Kisspeptin and RFamide-related peptide (RFRP) expression and terminal connections to gonadotropin-releasing hormone neurons in the brain: a novel medium for seasonal breeding in the sheep. Endocrinology.

[B083] Smith JT, Rao A, Pereira A, Caraty A, Millar RP, Clarke IJ (2008). Kisspeptin is present in ovine hypophysial portal blood but does not increase during the preovulatory luteinizing hormone surge: evidence that gonadotropes are not direct targets of kisspeptin in vivo. Endocrinology.

[B084] Smith JT, Li Q, Yap KS, Shahab M, Roseweir AK, Millar RP, Clarke IJ (2011). Kisspeptin is essential for the full preovulatory LH surge and stimulates GnRH release from the isolated ovine median eminence. Endocrinology.

[B085] Smith JT, Young IR, Veldhuis JD, Clarke IJ (2012). Gonadotropin-inhibitory hormone (GnIH) secretion into the ovine hypophyseal portal system. Endocrinology.

[B086] Sonstegard T, Fahrenkrug S, Carlson D (2017). Precision animal breeding to make genetically castrated animals for improved animal welfare and alternative breeding applications. J Anim Sci.

[B087] Spergel DJ (2019). Neuropeptidergic modulation of GnRH neuronal activity and GnRH secretion controlling reproduction: insights from recent mouse studies. Cell Tissue Res.

[B088] Thompson EL, Patterson M, Murphy KG, Smith KL, Dhillo WS, Todd JF, Ghatei MA, Bloom SR (2004). Central and peripheral administration of kisspeptin-10 stimulates the hypothalamic-pituitary-gonadal axis. J Neuroendocrinol.

[B089] Thorson JF, Desaulniers AT, Lee C, White BR, Ford JJ, Lents CA (2015). The role of RFamide-related peptide 3 (RFRP3) in regulation of the neuroendocrine reproductive and growth axes of the boar. Anim Reprod Sci.

[B090] Thorson JF, Heidorn NL, Ryu V, Czaja K, Nonneman DJ, Barb CR, Hausman GJ, Rohrer GA, Prezotto LD, McCosh RB, Wright EC, White BR, Freking BA, Oliver WT, Hileman SM, Lents CA (2017). Relationship of neuropeptide FF receptors with pubertal maturation of gilts. Biol Reprod.

[B091] Thorson JF, Prezotto LD, Adams H, Petersen SL, Clapper JA, Wright EC, Oliver WT, Freking BA, Foote AP, Berry ED, Nonneman DJ, Lents CA (2018). Energy balance affects pulsatile secretion of luteinizing hormone from the adenohypophesis and expression of neurokinin B in the hypothalamus of ovariectomized gilts†. Biol Reprod.

[B092] Thorson JF, Prezotto LD, Cardoso RC, Sharpton SM, Edwards JF, Welsh TH, Riggs AC, Amstalden M, Gary GL (2014). Hypothalamic distribution, adenohypophyseal receptor expression, and ligand functionality of rfamide-related peptide 3 in the mare during the breeding and nonbreeding seasons. Biol Reprod.

[B093] Tomikawa J, Homma T, Tajima S, Shibata T, Inamoto Y, Takase K, Inoue N, Ohkura S, Uenoyama Y, Maeda K, Tsukamura H (2010). Molecular characterization and estrogen regulation of hypothalamic KISS1 gene in the pig. Biol Reprod.

[B094] Topaloglu AK, Reimann F, Guclu M, Yalin AS, Kotan LD, Porter KM, Serin A, Mungan NO, Cook JR, Ozbek MN, Imamoglu S, Akalin NS, Yuksel B, O’Rahilly S, Semple RK (2009). TAC3 and TACR3 mutations in familial hypogonadotropic hypogonadism reveal a key role for Neurokinin B in the central control of reproduction. Nat Genet.

[B095] Treen AK, Luo V, Belsham DD (2016). Phoenixin activates immortalized GnRH and kisspeptin neurons through the novel receptor GPR173. Mol Endocrinol.

[B096] Tsutsumi R, Webster NJG (2009). GnRH pulsatility, the pituitary response and reproductive dysfunction. Endocr J.

[B097] Weems PW, Lehman MN, Coolen LM, Goodman RL, Litwack G (2018). Vitamins and hormones.

[B098] Wójcik-Gładysz A, Polkowska J (2006). Neuropeptide Y--a neuromodulatory link between nutrition and reproduction at the central nervous system level. Reprod Biol.

[B099] Wu M, Dumalska I, Morozova E, Van Den Pol AN, Alreja M (2009). Gonadotropin inhibitory hormone inhibits basal forebrain vGluT2-gonadotropin-releasing hormone neurons via a direct postsynaptic mechanism. J Physiol.

[B100] Wylot B, Tworus K, Okrasa S (2013). the effects of mu-, delta- and kappa-opioid receptor activation on luteinizing and follicle-stimulating hormone secretion from porcine pituitary cells. J Physiol Pharmacol.

[B101] Xu G, Li J, Zhang D, Su T, Li X, Cui S (2021). HSP70 inhibits pig pituitary gonadotrophin synthesis and secretion by regulating the corticotropin-releasing hormone signaling pathway and targeting SMAD3. Domest Anim Endocrinol.

[B102] Yang G, Su J, Yao Y, Lei Z, Zhang G, Li X (2010). The regulatory mechanism of neuromedin S on luteinizing hormone in pigs. Anim Reprod Sci.

[B103] Yang G, Su J, Yao Y, Lei Z, Zhang G, Liu Y, Liu J, Li X (2012). Distribution of neuromedin S and its receptor NMU2R in pigs. Res Vet Sci.

[B104] Yano T, Iijima N, Kakihara K, Hinuma S, Tanaka M, Ibata Y (2003). Localization and neuronal response of RFamide related peptides in the rat central nervous system. Brain Res.

[B105] Yoshida H, Habata Y, Hosoya M, Kawamata Y, Kitada C, Hinuma S (2003). Molecular properties of endogenous RFamide-related peptide-3 and its interaction with receptors. Biochim Biophys Acta..

[B106] Yosten GLC, Lyu R-M, Hsueh AJW, Avsian-Kretchmer O, Chang J-K, Tullock CW, Dun SL, Dun N, Samson WK (2013). A novel reproductive peptide, phoenixin. J Neuroendocrinol.

[B107] Zhou D, Zhuo Y, Che L, Lin Y, Fang Z, Wu D (2014). Nutrient restriction induces failure of reproductive function and molecular changes in hypothalamus–pituitary–gonadal axis in postpubertal gilts. Mol Biol Rep.

[B108] Zmijewska A, Czelejewska W, Dziekonski M, Gajewska A, Franczak A, Okrasa S (2020). Effect of kisspeptin and RFamide-related peptide-3 on the synthesis and secretion of LH by pituitary cells of pigs during the estrous cycle. Anim Reprod Sci.

